# A chloramine-based antiseptic polymer improves *Candida auris* skin decolonization compared to chlorhexidine in a murine model

**DOI:** 10.1128/aac.00713-26

**Published:** 2026-08-21

**Authors:** Janet Herrada, Lisa Long, Erin Marie San Valentin, Fnu Samiksha, Nicole Byrnes-Shaver, Lynne Harding, Thomas S. McCormick, Mahmoud Ghannoum

**Affiliations:** 1 Department of Dermatology, School of Medicine, Case Western Reserve University 2546, Cleveland, Ohio, USA; 2 iFyber, LLC, Ithaca, New York, USA; University Children's Hospital Münster, Münster, Germany

**Keywords:** skin decolonization, antifungal, *Candida auris*, chlorhexidine gluconate

## Abstract

*Candida auris* frequently demonstrates resistance to multiple antifungal classes and has been classified as a critical priority pathogen by the World Health Organization and the first fungal pathogen designated an urgent threat by the U.S. Centers for Disease Control and Prevention. Patients with skin colonization face a 7%–20% risk of invasive infection, with 30-day mortality of 30%–72%. While chlorhexidine gluconate (CHG) is widely used for general skin decolonization, it fails to reliably clear *C. auris* from patient skin, and no proven decolonization protocol exists. We evaluated a novel chloramine-based antiseptic polymer, polyvantoin chlorine (AVA-003). *In vitro* CLSI broth microdilution demonstrated activity against isolates from all four major *C. auris* clades (I–IV), with minimum inhibitory concentrations ranging from 250 to 500 µg/mL. Two proof-of-concept experiments using a validated murine model assessed *in vivo* efficacy. Female BALB/c mice were inoculated with clinical isolate *C. auris* MRL-35368 and treated with 1.2% AVA-003, 2% CHG, or vehicle controls across two phases examining formulation and treatment duration. AVA-003 reduced fungal burden at both ear and dorsal sites compared to untreated and CHG-treated controls. Specifically, 1.2% AVA-003 reduced ear burden versus untreated controls by 3.07 log colony-forming units (CFU)/g (*P* = 0.003, phase I) and versus 2% CHG at 3 days by 1.42 log CFU/g (*P* < 0.0001, phase II); dorsal burden was reduced by 3.58 log CFU/g versus untreated controls (*P* < 0.0001). In phase I, 1.2% AVA-003 achieved undetectable burden (0 CFU/g) at both sites. In phase II, AVA-003 significantly reduced but did not uniformly eradicate *C. auris* burden. Its advantage over CHG was greater at 3 days than 7 days, likely reflecting progressive dorsal hair regrowth, a recognized limitation of the murine model. These findings support AVA-003 as a promising *C. auris* decolonization candidate warranting further clinical investigation.

## INTRODUCTION

*Candida auris* (also known as *Candidozyma auris*) is an emerging multidrug-resistant yeast of increasing concern, with many strains exhibiting multidrug resistance and some displaying pan-resistance (i.e., resistant to all clinically available antifungals). Consequently, this fungal pathogen is classified as a critical priority by the World Health Organization (WHO) and the first fungal pathogen to be listed by the U.S. Centers for Disease Control and Prevention (CDC) as an urgent threat (1–4). Although overall case counts remain relatively low compared to other *Candida* species, the incidence of *C. auris* clinical cases has increased substantially in the United States, with a nearly three-fold rise in cases between 2019 and 2021 (5), underscoring the trajectory of concern for healthcare systems. For approximately 95% of patients, *C. auris* colonization involves only the skin, where *C. auris* often forms robust multilayer biofilms, typically on the outer epidermal layers, without invading deeper tissues (6–10). From the skin, *C. auris* can rapidly shed into the environment, persist on human skin and abiotic surfaces for extended periods, and resist or rebound quickly from routine disinfection used in healthcare settings (2, 11). This makes for easy transmission through close contact with colonized patients, contact with contaminated hospital surfaces (including bedrails, bedside tables, door handles, toilets, and medical equipment), or skin-to-skin contact with infected individuals (2, 11–16). Immunocompromised patients or those with significant medical comorbidities, indwelling medical devices (e.g., vascular catheters, G-tubes, and endotracheal tubes), or accidental wounds or cuts are more likely to develop invasive fungal infections (9, 17). Importantly, mortality rates of patients with systemic *C. auris* infection range between 30% and 72% in 30 days (6, 18–20).

Among *C. auris* strains, 87%–100% are resistant to fluconazole; 8%–35% are resistant to amphotericin B; 0%–8% are resistant to echinocandins; and over 40% express combined resistance to two or more classes of antifungals (2, 6, 18). Echinocandin resistance, particularly when combined with resistance to azole and amphotericin B (pan-resistance), is a significant clinical and public health concern (2). *C. auris* has the potential to become more resistant in the course of a treatment with antifungal drugs, with approximately three times the number of pan-resistant cases in 2021 compared to the previous 2 years (5, 21). Thus, developing novel, effective antifungals that target pan-resistant strains, as well as antiseptics that can decolonize the human skin, is critically needed.

Currently, there are no official guidelines for *C. auris* decolonization (11, 22). Sodium hypochlorite (NaOCl) and povidone iodine have been evaluated for use against *C. auris* without much success (11, 23). Recent clinical evidence has also demonstrated that nasal iodophor application can reduce *C. auris* nasal carriage in nursing home residents (10, 24). Although chlorhexidine gluconate (CHG) is the most widely used agent for *C. auris* decolonization attempts, its clinical efficacy is variable. A recent study by Proctor et al. (25) demonstrated that residual CHG in concentrations of 625 µg/mL was associated with meaningful reductions in *C. auris* bioburden in a healthcare setting. However, other clinical studies also continue to show persistent colonization despite daily topical application of CHG (8, 10, 26, 27). Systemic antifungal therapies have not achieved reliable decolonization. They are, therefore, not recommended for colonization in the absence of invasive infection, particularly given the risk of selecting for resistance (28, 29). The CDC recognizes that there is a desperate need to develop a treatment to decolonize *C. auris* to lower the overall mortality and mitigate the need for contact precautions (30).

A novel antiseptic, polyvantoin chlorine (AVA-003), which serves as a delivery mechanism of hypochlorous acid for topical use, has been developed, allowing sustained antimicrobial activity at a concentration tolerable and safe to the skin. Polyvantoin chlorine is a polymer that stabilizes oxidative chlorine and is similar in concept to povidone iodine, where povidone stabilizes oxidative iodine. Given the popularity of hypochlorous acid as a biocompatible skin cleanser and the similarity in oxidative chemistry to povidone iodine, we hypothesize that AVA-003 could achieve decolonization of *C. auris* on the skin (20). In this study, we aimed to compare the efficacy of AVA-003 to CHG and investigated the topical safety with the daily application in an *in vivo* mouse model.

## RESULTS

### *In vitro* antifungal activity of 1.2% AVA-003

The *in vitro* antifungal activity of AVA-003 against all four major *C. auris* clades is summarized in Table 1. Minimum inhibitory concentration (MIC) values ranged from 250 µg/mL for clade I (AR-0381) and clade II (AR-0382) isolates to 500 µg/mL for clade III (AR-0384) and clade IV (AR-0385) isolates. Notably, minimum fungicidal concentration (MFC) values were equal to MIC values across all tested isolates, indicating that AVA-003 is fungicidal rather than fungistatic against *C. auris* at the inhibitory concentration. Results were reproducible across both experimental replicates. The consistent activity across all four genetically distinct clades is clinically relevant, as clade-specific differences in cell surface composition have been shown to influence susceptibility to other antiseptic agents. These findings provided the mechanistic rationale to advance AVA-003 into the *in vivo* murine decolonization model.

**TABLE 1:** *In vitro* antifungal activity of AVA-003 against *C. auris^a^*

Species	FTL no.	Clade	AVA-003 (μg/mL)	
			MIC	MFC
*Candida auris*	AR-0381	I	250	250
	AR-0382	II	250	250
	AR-0384	III	500	500
	AR-0385	IV	500	500

^*a*^ Minimum inhibitory concentration (MIC) and minimum fungicidal concentration (MFC) values represent the consensus of two independent experimental replicates, each performed in six technical replicates per isolate.

For the *in vivo* murine model experiments, both phase I and phase II studies consisted of *n* = 5 mice per treatment group. We acknowledge that this sample size was chosen as appropriate for proof-of-concept work and limits statistical power, particularly given the variability in fungal burden observed across animals.

### Dose finding and protocol development experiment (phase I)

**Fig 1:**
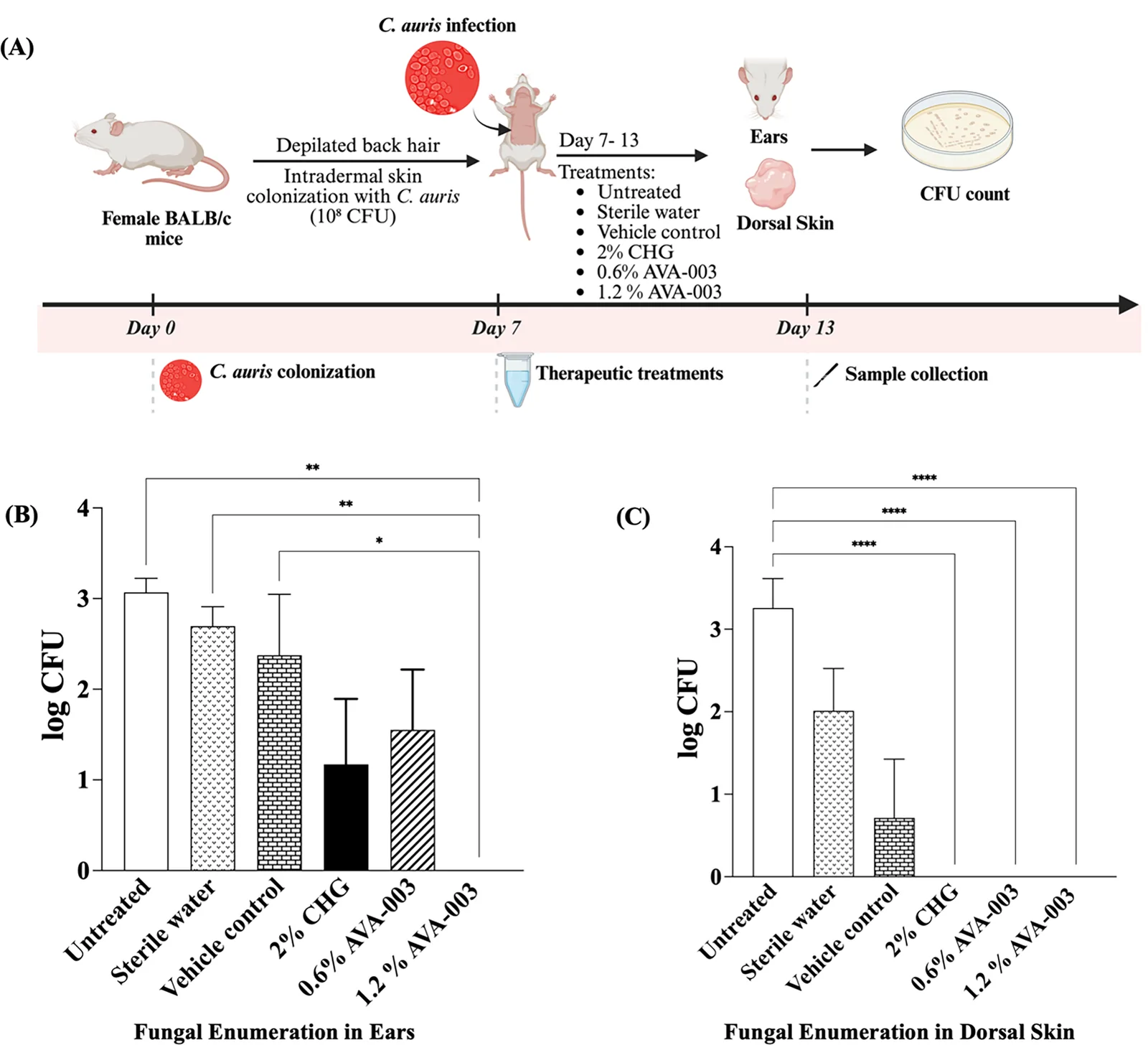
(**A**) Schematic representation of the experimental design for comparison of formulation. The hair on mice’s dorsal skin was trimmed, depilated with Nair 2 days before treatment, and infected with *C. auris* on days 2, 4, and 6, including the ears. (**B**) Fungal enumeration in ears of mice after 7 days of treatment with different formulations. (**C**) Fungal enumeration in the dorsal skin of mice following a 7-day treatment with different formulations. Data show mean log colony forming units (CFU) ± standard error. Statistical significance was determined using one-way analysis of variance followed by a Tukey’s multiple comparison analysis. *, *P* ≤ 0.05; **, *P* ≤ 0.01; ****, *P* ≤ 0.0001.

Different treatment formulations led to varying levels of *C. auris* fungal burden on the ears and dorsal surface of mice over 7 days of treatment. The schematic representation of the experiment is shown in Fig. 1A. Overall, 0.6% and 1.2% AVA-003 seemed to reduce *C. auris* colonization in a site-dependent manner (Fig. 1).

The untreated control group had a mean fungal load of 3.07 ± 0.34 log colony-forming units (CFU)/g on the ears. Treatment with 0.6% AVA-003 resulted in a mean fungal burden of 1.55 ± 1.49 log CFU/g, which did not differ significantly from the untreated control (*P* = 0.295) or 2% CHG (*P* = 0.994) (Fig. 1B). In contrast, 1.2% AVA-003 significantly reduced ear fungal burden to 0 CFU/g compared to untreated controls (*P* = 0.003). No significant differences were detected between 1.2% AVA-003 and 0.6% AVA-003 (*P* = 0.274) or between 2% CHG and untreated controls (*P* = 0.113).

On the other hand, the dorsal skin of mice in the untreated group showed an average fungal load of 3.26 ± 0.80 log CFU/g. Treatment with sterile water (2.01 ± 1.14 log CFU/g, *P* = 0.241) did not significantly reduce fungal load compared to the untreated control (Fig. 1C). In contrast, a decrease in fungal burden was observed with 2% CHG, 0.6% AVA-003, and 1.2% AVA-003, with fungal burden falling below the lower limit of detection (10 CFU) in all animals in these groups. All three treatments demonstrated significantly lower fungal burden compared to the untreated control (*P* < 0.001). No significant differences were detected among the three active treatment groups (*P* > 0.05).

Based on these findings, we further investigated the effects of 1.2% AVA-003 on *C. auris* colonization of mice in 3- and 7-day treatments (phase II).

### AVA-003 achieves superior *C. auris* decolonization over 2% CHG following a 3-day regimen (phase II)

**Fig 2:**
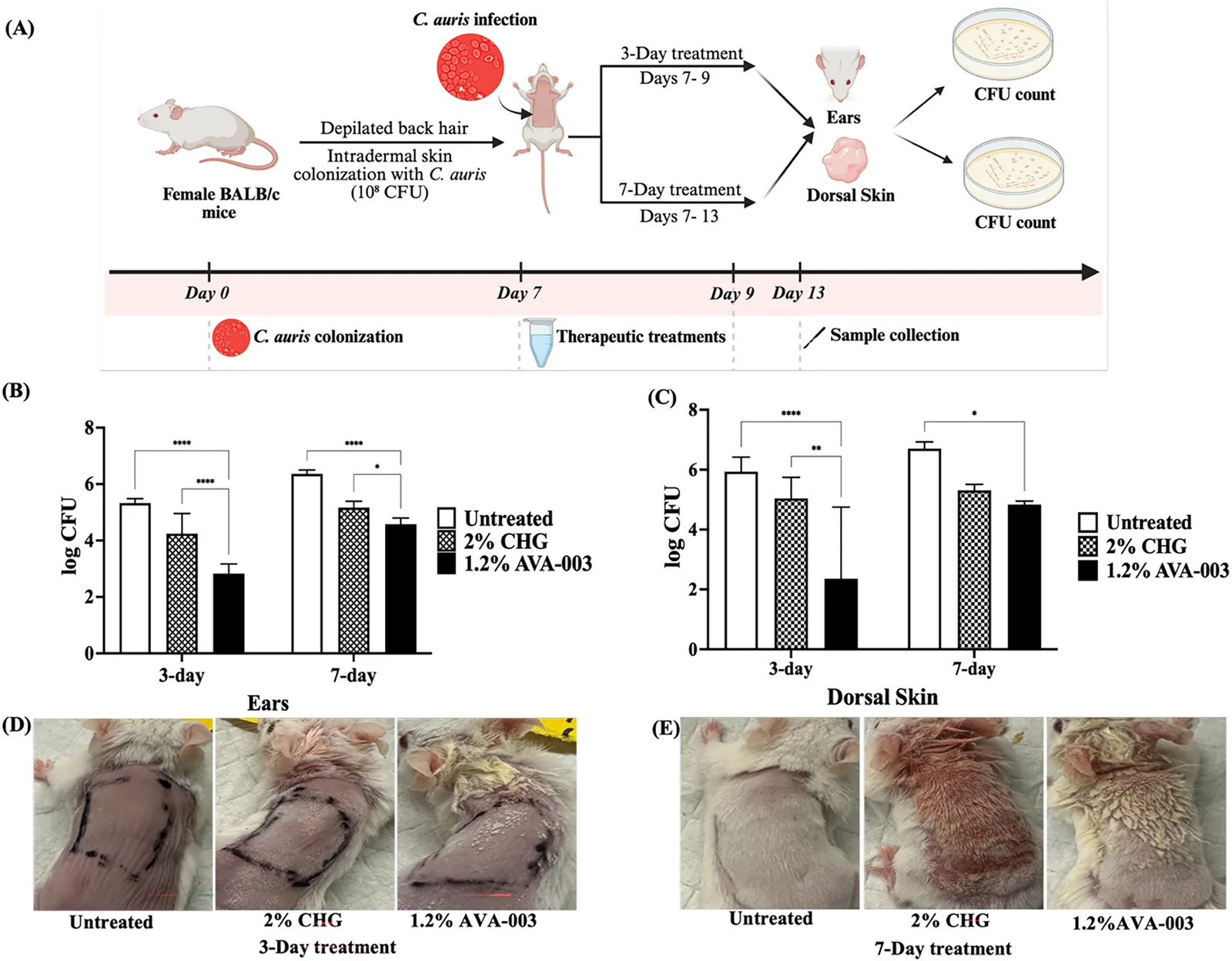
(**A**) Schematic representation of the experimental design for treatment duration comparison (3 days versus 7 days). Mice’s hair on the dorsal skin was trimmed, depilated with Nair 2 days before treatment, and infected with *C. auris* on days 2, 4, and 6, including the ears. Fungal load in the dorsal skin and ears of mice was determined on days 10 and 14 post-treatment. (**B**) On the ears, both 3- and 7-day treatments show significant reduction in fungal load of 1.2% AVA-003-treated mice compared to both untreated and 2% CHG controls. *, *P* < 0.05; ****, *P <* 0.0001. (**C**) On the dorsal skin at 3 days, a significant reduction in fungal load can be observed when comparing 1.2% AVA-003-treated mice to untreated controls and 2% CHG. **, *P* < 0.01; ****, *P <* 0.0001. However, a significant reduction in fungal load was only observed in the untreated control versus 1.2% AVA-003 at 7 days. *, *P* < 0.05. (**D** and **E**) Clinical appearance of treated mice inoculated with *C. auris* during 3- and 7-day treatment. Data are presented as mean ± standard error. Statistical significance was determined using one-way analysis of variance followed by Tukey’s test. *P* ≤ 0.05 was considered significant.

Similar to the first set of *in vivo* experiments, the groups of female BALB/c mice were infected with a clinical isolate of *C. auris* MRL #35368 strain at a dose of 1 × 10^8^ CFU per mouse. Ears and dorsal skin of mice were collected on days 10 and 14 post-treatment to enumerate the tissue fungal load. The schematic representation of the experimental design is shown in Fig. 2A.

At the end of each treatment period, tissues from the dorsal skin and ears of the mice were collected, and fungal burden was quantified as log CFU per gram of tissue. In a 3-day treatment, the untreated control group showed the highest fungal burden (5.33 ± 0.2 log CFU/g and 5.94 ± 0.5 log CFU/g) in both the ear (Fig. 2B) and dorsal skin (Fig. 2C) of mice, respectively. The 2% CHG group showed a fungal burden of 4.24 ± 0.7 log CFU/g and 5.04 ± 0.7 log CFU/g on the ear and dorsal skin, respectively, while the 1.2% AVA-003 showed the lowest fungal burden of 2.83 ± 0.3 log CFU/g and 2.35 ± 2.4 log CFU/g on the ear and dorsal skin, respectively (Fig. 2).

For the 7-day treatment, fungal burdens in the dorsal skin are shown in Fig. 2C. The untreated control group showed the highest fungal burden (6.70 ± 0.2 log CFU/g and 6.36 ± 0.1 log CFU/g in the dorsal skin and ear, respectively). The 2% CHG group showed a fungal burden of 5.31 ± 0.2 log CFU/g (on the dorsal skin) and 5.17 ± 0.2 log CFU/g (on the ears), while the 1.2% AVA-003 showed the lowest fungal burden of 4.83 ± 0.1 log CFU/g (on the dorsal skin) and 4.58 ± 0.2 log CFU/g (on the ears).

A two-way analysis of variance (ANOVA) was performed to evaluate the independent and the combined effects of treatment group and duration on *C. auris* fungal burden. Analysis of the ear samples showed a significant treatment × duration interaction (*P* = 0.0340), duration main effects (*P* < 0.0001), and treatment main effects (*P* < 0.0001). Tukey’s post hoc comparison showed that AVA-003 treatment significantly reduced fungal burden compared to untreated controls at both time points (*P* < 0.0001) and compared to 2% CHG (3-day: *P* < 0.0001, 7-day: *P* = 0.0400) on the ear.

In contrast, no significant treatment × duration interaction (*P* = 0.0655) was detected for the dorsal tissue samples, indicating that the relative efficacy of each treatment was maintained regardless of whether mice were treated for 3 or 7 days. Analysis of main effects for dorsal tissues also revealed significant effects of treatment group (*P* < 0.0001) and duration (*P* = 0.0053). Tukey’s post hoc comparison similarly showed that 1.2% AVA-003 significantly reduced fungal burden in dorsal tissues compared to untreated controls (*P* < 0.0001) and 2% CHG (*P* = 0.0013) at the 3-day treatment, but showed significant reduction (*P* = 0.0245) of fungal burden only in 1.2% AVA-003 compared to untreated at 7 days.

Throughout the phase II treatment period, daily clinical observation of all mice did not show any visible adverse effects on the skin at either anatomical site. However, two mice from the 1.2% AVA-003 group exhibited minor skin dryness of the ears and dorsal skin near the end of the 7-day treatment. No visible skin changes, including erythema, erosion, or dryness, were observed in the 2% CHG-treated animals during either treatment phase, allowing direct tolerability comparison between the two agents.

## DISCUSSION

In this study, we demonstrated the activity of the novel chloramine-based antiseptic polymer, AVA-003, in both *in vitro* and *in vivo* experiments. Before advancing to the murine models, we first characterized the antifungal spectrum of AVA-003 *in vitro* using standardized CLSI broth microdilution methodology. AVA-003 demonstrated consistent inhibitory activity against all four genetically distinct *C. auris* clades tested (clade I [AR-0381], clade II [AR-0382], clade III [AR-0384], and clade IV [AR-0385]), with MIC values ranging from 250 to 500 µg/mL. This lack of variation across different clades is of clinical importance, given the well-documented variation in susceptibility between clades to systemic antifungal treatments (31–33).

The *in vitro* activity of AVA-003 seemed to be consistent with the non-specific oxidative mechanism, which targets proteins and essential enzymes common to all fungi rather than a pathway or structure-specific target utilized by antifungals such as azoles or echinocandins that allows for resistance development through pathway mutations. The consistent activity of AVA-003 across all four *C. auris* clades is a significant advantage. Clade-specific differences in cell surface composition and gene expression have been shown to influence susceptibility to other antiseptics, making pan-clade efficacy a desirable property for a decolonization agent intended for use in healthcare settings where the infecting clade may be unknown. These *in vitro* findings provided the mechanistic rationale and the confidence to advance AVA-003 into the *in vivo* murine decolonization model.

AVA-003 (1.2%) was tested against 2% CHG in a mouse model for *C. auris*. Two percent CHG was selected as a comparator because it is the agent most commonly employed in *C. auris* containment protocols in healthcare settings despite the absence of an established decolonization standard and its recognized limited efficacy against *Candida* species (11). The comparison is intended to contextualize AVA-003 performance against current practice, not to imply CHG represents a validated decolonization benchmark. In phase I and phase II experiments conducted on the post-auricular and dorsal skin of the mice with different treatment durations and formulations, 1.2% AVA-003 demonstrated potential to substantially reduce *C. auris* skin burden. In both phases of *in vivo* experiments, 1.2% AVA-003 demonstrated superior performance compared to untreated controls and, at the 3-day time point, significantly outperformed 2% CHG at both anatomical sites. This differential effect was attenuated at 7 days, likely reflecting progressive dorsal hair regrowth interfering with product contact, which is a recognized limitation of the murine model rather than loss of drug activity. Additionally, applying the combined 1.2% AVA-003 formulation once per day did not result in any skin irritation on mice.

The enhanced efficacy of 1.2% AVA-003 likely stems from its unique chemical design. AVA-003 is a polymer containing an N-chloramine monomer copolymerized with a sodium sulfonate functionalized acrylamide monomer. AVA-003 stabilizes chlorine, like povidone stabilizes iodine, resulting in reduced skin irritation and reduced neutralization in the presence of organic material. The stabilization of chlorine enables AVA-003 to survive with the chlorine intact after drying from solution. This chlorine retention, combined with the broad-spectrum oxidative chemistry of chlorine and the surfactant activity of the sulfonate monomer, likely provides the sustained and multimodal antimicrobial action necessary to achieve *C. auris* decolonization from the skin. Unlike the biguanide mechanism of CHG, which relies on membrane disruption and can be compromised by pH and non-specific binding (34, 35) to organic materials (e.g., blood, proteins, and sebum, etc.), AVA-003 employs a dual-action approach. First, the chlorine acts as a broad-spectrum oxidant that denatures the proteins and essential enzymes. Second, the sulfonate monomer provides the surfactant properties that likely reduce the ability of *C. auris* to adhere to surfaces. Gussin et al. (36) recently confirmed that *C. auris* rapidly sheds onto surfaces and the environment, particularly in clinical settings. They also showed that CHG can effectively reduce *C. auris* shedding when residual concentrations are sufficiently maintained at high concentrations (≥156 µg/mL). Our findings with AVA-003 are critical because achieving superior skin decolonization in the murine model suggests that AVA-003 may also overcome the concentration gap that limits the effectiveness of CHG.

In the first phase of testing in mice, good results were obtained from inoculation of the ears of the mouse model. The 1.2% AVA-003 treatment was superior to 2% CHG and statistically different from the untreated. Moreover, no irritation or adverse effects were observed on any animals tested with 1.2% AVA-003 at this concentration for 7 days, even with dual application of 1.2% AVA-003 liquid and gel. However, the overall control pathogen burden was lower (<3.5 log CFU/g) than anticipated, which makes definitive conclusions difficult. For the dorsal tissues of the animals, plastic dressings were applied, but not to the ears. This protocol may have caused challenges with recovery from the dorsal areas of the mice because the plastic covering may have aided in removing *C. auris*, even for treatments with sterile water. There was also concern that the regrowth of hair might be an issue with greater hair density on the dorsum of the mice as opposed to the ears. The use of the plastic covering was eliminated, and in the subsequent testing round, liquids were subsequently applied with a wetted sterile gauze and allowed to dry. Likewise, inoculum was applied more thoroughly and for a longer time to encourage higher pathogen loads.

In the phase II *in vivo* experiments, treatment duration was shortened to 3 or 7 days to minimize potential confounding effects of dorsal hair regrowth, which required a reduction in the number of treatment groups. Accordingly, three conditions were compared across both durations: untreated control, 2% CHG, and 1.2% AVA-003. Results were more consistent and robust than the phase I experiments, with AVA-003 demonstrating a clear difference over 2% CHG in reducing *C. auris* burden at both sites at 3 days. The greatest reduction was observed on the dorsal skin following 3-day treatment, where AVA-003 achieved a 3.58 log CFU/g reduction compared to untreated controls and a 2.68 log CFU/g reduction compared to 2% CHG. No skin irritation or adverse effects were observed in any animal at either treatment duration.

The 3-day treatment produced greater reductions than the 7-day treatment, which may reflect progressive hair regrowth at the dorsal tissue interfering with product contact and retention over the longer treatment period. This can then be considered a limitation of the murine model rather than a loss of drug activity. Due to animals with similar treatment being cohorted together, the cages from different cohorts being close together, and the bedding and cages of all groups not being disinfected after initial inoculation until the experiment was finished, complete elimination of *C. auris* in this model was not expected.

In both phase I and phase II *in vivo* testing, AVA-003 showed significant reductions compared to control, and in most rounds compared to 2% CHG. Notably, AVA-003 was evaluated without additives or excipients, whereas the CHG comparator was a fully formulated commercial product, suggesting that formulation optimization could yield further improvements in efficacy. Together, these results indicate that AVA-003 treatment is superior to 2% CHG and should be assessed further for *C. auris* decolonization.

There are several limitations to consider when interpreting these findings. First, the treatment groups only consisted of *n* = 5 mice, limiting statistical power; non-significant comparisons may thus reflect insufficient power rather than absence of effect. Second, the phase I control burden was lower than anticipated (<3.5 log CFU/g), limiting the efficacy conclusions. Third, the study lacked comparator arms: phase II lacked a vehicle-only hydrogel control arm to address the polymer contribution from the oxidative chlorine component, and no hypochlorous acid-only comparator was included to distinguish the effect of the chlorine chemistry itself. Fourth, only one fungal pathogen was evaluated; extension to other multidrug-resistant organisms should be conducted. Fifth, the tolerability of 1.2% AVA-003 in humans was also not assessed in this study; formal dermal safety evaluation is required before clinical application. In addition, *C. auris* also colonizes the nares, a site implicated in transmission and used for clinical surveillance. The present study evaluated topical dermal application only; extending AVA-003 to an intranasal formulation would require dedicated tolerability and safety assessment and warrants future investigation.

### Conclusions

*C. auris* represents an escalating threat to healthcare systems globally, and the lack of an effective decolonization strategy remains unmet. In this study, we demonstrated preliminary evidence that 1.2% AVA-003, polyvantoin chlorine, exhibits antifungal activity *in vitro*, including consistent activity against all four *C. auris* clades. AVA-003 also shows superior activity compared to 2% CHG in a murine model of *C. auris* skin colonization across two different treatment durations and anatomical sites. The efficacy of AVA-003 is likely attributable to its multimodal mechanism, which combines residual oxidative chlorine activity with surfactant disruption of fungal adhesion. These are properties that are distinct from the biguanide mechanism of CHG. No safety concerns were observed at the concentrations tested in the murine model; human skin tolerability was not assessed in this study, and formal dermal safety evaluation will be an essential next step prior to clinical applications. While further refinement of the animal model and transition to a human skin model are needed, based on the limited data presented, these findings provide a preliminary rationale for continued preclinical development of 1.2% AVA-003 as a candidate decolonization agent for *C. auris*.

## MATERIALS AND METHODS

### *C. auris* strains and culture conditions

Four *C. auris* strains representing clades I, II, III, and IV obtained from iFyber, LLC (Ithaca, New York) were used to evaluate the antifungal activity of AVA-003 *in vitro*. Each strain was tested with six technical replicates across two independent experiments. For *in vivo* testing, a clinical isolate of *C. auris* (MRL #35368, clade I/ South Asian clade), obtained from the Center for Medical Mycology, University Hospitals Cleveland Medical Center (Cleveland, OH), was used as the skin colonizing strain. *C. auris* was stored in 25% glycerol at −80°C and revived on Sabouraud Dextrose Broth (Difco Laboratories, Detroit, MI) by growing at 37°C for 48 hours.

### Test materials

Liquid and hydrogel formats of chloramine-based antiseptic polymer, polyvantoin chlorine (AVA-003), and non-biocidal vehicle hydrogel were supplied by AvantGuard, Inc. (Ithaca, NY) and stored at 4°C until use. CHG (2% Hibiclens) was purchased from Mölnlycke Health Care (Norfolk, GA). Sterile water served as controls.

### *In vitro* antifungal susceptibility testing

MIC and MFC were determined by the CLSI broth microdilution method (M27) (37). Briefly, a stock solution of 1.2% AVA-003 was prepared in sterile water and diluted further in RPMI 1640 (with phenol red and 0.2% glucose, without sodium bicarbonate) buffered with 0.165 M MOPS (pH 7.0). The concentration range tested was 1.95–1,000 μg/mL. MIC was defined as the lowest concentration yielding an OD_650_ <0.1 relative to drug-free growth controls (complete media + corresponding *C. auris* strain only).

To determine the MFC of AVA-003, wells showing no visible growth were cultured on Sabouraud Dextrose Agar (SDA, Difco Laboratories) and incubated at 37°C for 24 hours. MFC was defined as the lowest concentration achieving ≥99.9% (a minimum of 3 log_10_) reduction in viable organisms.

### *In vivo* murine colonization model

#### Animal husbandry and ethics

The *in vivo* experiments were performed using female BALB/c mice (Charles River Laboratories, Wilmington, MA), provided by the Animal Resource Center at Case Western Reserve University. The mice were subjected to a 12:12 light-dark cycle with controlled temperature (16°C–22°C) and relative humidity to ensure species-appropriate conditions and were acclimated for a minimum of 5 days prior to the experiment. The animals were euthanized using carbon dioxide narcosis.

#### Inoculum preparation

For the inoculum preparation, *C. auris* was plated on SDA and incubated at 37°C for 48 hours. *C. auris* cells were then harvested by centrifugation at 1,450 × *g* for 5 minutes, followed by three normal saline (0.85% NaCl) washes and a final resuspension in 5 mL PBS (1×). The cells were counted using a hemacytometer, and an inoculum of about 10⁸ cells/mL was prepared using 1× PBS. To verify the inoculum count, 10-fold serial dilutions of the *C. auris* working yeast cell suspensions were prepared in PBS and plated onto SDA. The plates were incubated at 37°C for 48 ± 4 h, and the colony counts were determined.

#### Skin colonization procedure

The colonization of mice with *C. auris* was done following a previously described method by Ghannoum et al. (38). Briefly, 2 days before infection, the dorsal hair of the mice was trimmed and chemically depilated with Nair hair removal cream (Church & Dwight Co., Inc., Ewing, NJ). On day 0, a 1.5 cm² stencil square was used to mark the target area of the dorsal surface of mice. To disrupt the skin and induce yeast colonization, a strip of autoclave tape was applied to the target area on the dorsal surface of the mouse. The tape was left on for around five seconds before being removed; this procedure was repeated four times. A cotton-tipped applicator soaked with *C. auris* inoculum (10^8^ CFU/mL) was applied to the designated area until the entire square was visibly moist (five swabs). In addition, *C. auris* inoculum (10^8^ CFU/mL) was administered to both the pinnae of the ears with a moistened cotton tip applicator (five rubs on each ear). This inoculation protocol was repeated on days 2, 4, and 6 to establish robust colonization.

#### Phase I: dose-finding and protocol-development experiment

Phase I served primarily as a dose-finding and protocol-development experiment. Different doses of AVA-003, as well as the effect of the vehicle controls, were examined. Beginning on the seventh day post-inoculation, mice were randomized into six treatment groups (*n* = 5 per group, Fig. 1): untreated control, sterile water, sterile water plus vehicle hydrogel, 2% CHG, 0.6% AVA-003 liquid plus 0.3% AVA-003 hydrogel, and 1.2% AVA-003 liquid plus 1.2% AVA-003 hydrogel. Treatments were administered once a day for 7 days (days 7–13). For the liquid treatments, sterile gauze was soaked in test materials (sterile water, 2% CHG, and AVA-003 liquids) and applied over the dorsal area of the mice. The area was then covered with polyurethane film dressings (Tegaderm; 3M, St. Paul, MN). After 10 minutes, the dressing was removed. For the hydrogel-treated groups, the hydrogel was then applied without delay, spread across the target area, and allowed to absorb.

To treat the ears, liquids were applied using a cotton swab soaked with test materials. Hydrogels were applied to the ears in the same fashion as in the back after the ears were dried for about 10 minutes. Mice were euthanized by CO_2_ inhalation on day 14. For CFU quantification, a 10 mm biopsy punch was collected from the colonized area on the dorsal surface, and the right and left ears were aseptically harvested post-mortem.

#### Phase II: treatment duration optimization

Phase II differed from phase I in two key respects. First, based on phase I findings, only the optimal dose of AVA-003 (1.2% AVA-003 liquid plus 1.2% AVA-003 hydrogel) was used and evaluated at two time points (3 and 7) alongside 2% CHG and untreated controls. Second, the Tegaderm polyurethane film dressing was omitted from the liquid application protocol, as occlusive dressing was identified as a potential contributor to the vehicle hydrogel effect observed on dorsal skin in phase I. All the other colonization and application procedures were identical to those described in sections 2.4.3 and 2.4.4. Mice colonized with *C. auris* (as described above) were randomized into six groups (*n* = 5 per group) in a 3 × 2 design, with three treatment conditions (untreated control, 2% CHG, and 1.2% AVA-003 liquid plus 1.2% AVA-003 hydrogel) and two treatment durations (3 and 7 days). At the time of euthanasia, dorsal skins (10 mm biopsy punch) and bilateral ear tissues were collected from all animals for CFU quantification.

### Statistical analysis

Data were shown as means ± standard error. Statistical significance was determined using one- or two-way ANOVA followed by Tukey’s multiple comparison test using GraphPad Prism for macOS (v11.0.0 [93]). *P* ≤ 0.05 was considered significant.
